# Vibrational disorder and densification-induced homogenization of local elasticity in silicate glasses

**DOI:** 10.1038/s41598-021-04045-6

**Published:** 2021-12-27

**Authors:** Omar Benzine, Zhiwen Pan, Courtney Calahoo, Michal Bockowski, Morten M. Smedskjaer, Walter Schirmacher, Lothar Wondraczek

**Affiliations:** 1 Otto Schott Institute of Materials Research, University of Jena, 07743 Jena, Germany; 2 Institute of High-Pressure Physics, Polish Academy of Sciences, 01-142 Warsaw, Poland; 3 Department of Chemistry and Bioscience, Aalborg University, 9220 Aalborg, Denmark; 4 Institute of Physics, University of Mainz, 55099 Mainz, Germany

**Keywords:** Condensed-matter physics, Structure of solids and liquids, Characterization and analytical techniques, Glasses

## Abstract

We report the effect of structural compaction on the statistics of elastic disorder in a silicate glass, using heterogeneous elasticity theory with the coherent potential approximation (HET-CPA) and a log-normal distribution of the spatial fluctuations of the shear modulus. The object of our study, a soda lime magnesia silicate glass, is compacted by hot-compression up to 2 GPa (corresponding to a permanent densification of ~ 5%). Using THz vibrational spectroscopic data and bulk mechanical properties as inputs, HET-CPA evaluates the degree of disorder in terms of the length-scale of elastic fluctuations and the non-affine part of the shear modulus. Permanent densification decreases the extent of non-affine elasticity, resulting in a more homogeneous distribution of strain energy, while also decreasing the correlation length of elastic heterogeneity. Complementary ^29^Si magic angle spinning NMR spectroscopic data provide a short-range rationale for the effect of compression on glass structure in terms of a narrowing of the Si–O–Si bond-angle and the Si–Si distance.

## Introduction

Elasticity is a primary design target in modern glass science and technology^1,2^. For a variety of applications, it is often desirable to adapt glass formulations for enhanced shear and/or Young’s modulus and, thus, enhanced rigidity or stiffness^3^. For example, this may enable reduction of the wall thickness of a glass product in a given application and, hence, reduction of product weight and embodied CO_2_. To this end, the effect of glass composition on elastic moduli is often estimated to a satisfactory accuracy using semi-empirical mixing or regression models^4–9^. Most of these approaches start from the Makishima-Mackenzie (MM) model^5^, which remains today’s most popular approach for predicting Young’s modulus from a glass composition. It takes into account the dissociation energies of the oxide constituents in crystalline form, and the corresponding occupied volume as derived from ionic radii. However, the MM model as well as follow-up approaches are known to frequently underestimate^3,10,11^ or overestimate^12^ Young’s modulus. As often pointed-out, MM-type approaches perform best only for glasses with compositions close to a corresponding crystal of equivalent density, for which the ionic radii are precisely known^3^. Deviations from such model descriptions are due to disorder-related local variations of the structural cohesion^13^.

The existence of spatially heterogeneous elasticity is well established through in silico^14,15^ and experimental^16,17^ studies of simple glasses. ‘Soft’ and ‘hard’ domains were found to coexist across a typical length scale of a few nanometers (or a few tens of particles^18,19^). This heterogeneous nature is reflected in the observation that displacement fields on the microscopic level differ from macroscopic strain analysis, what has been described as *non-affine* displacement^20,21^. During non-affine deformation, individual particles undergo correlated displacements. The associated mesoscopic correlation length was found to be comparable to the one of elastic heterogeneity^15,22^. In effect, this heterogeneous response leads to a significant decrease in the effective elastic moduli (as compared to a classical *affine* approximation such as the Born–Huang theory^23^.

Several methods have been proposed to map the local elastic properties of simple model glasses, mostly by numerical simulation^14,24^, but real-world experimental data remain difficult to obtain. Proposed methods are sometimes ambiguous in terms of the assessed length scale (e.g.,^25^). An analytical framework for the evaluation of spatially fluctuating elastic constants is provided by the heterogeneous-elasticity theory (HET^26^). HET assumes that spatial disorder leads to random fluctuations in structural rigidity. On this assumption, it provides expressions for the vibrational density of states and enables predictions of the frequency-dependence of Raman scattering and of the temperature-dependence of the heat capacity in disordered solids^27^. In return, HET can be employed to quantify heterogeneous elasticity, non-affinity and the underlying length scales using experimental data such as Raman scattering spectra and/or low-temperature heat capacity for input^28^. In the present paper, we follow this approach to elucidate the effects of network compaction on heterogeneous elasticity in the archetypal soda-lime-magnesia silicate (SLMS) glass. SLMS is representative for the most common group of glass materials used in commodity products such as glass containers, windows, tableware or automotive windshields. We use HET as a means to assess elastic disorder in SLMS and investigate variations in the population density distribution (PDD) of the local shear modulus *P*(*G*) which occur as the fictive pressure of the glass increases to 2 GPa. From this, we extract pressure-induced changes in non-affine elasticity. The PDD is obtained from the coherent potential approximation of HET (HET-CPA) with THz Raman spectroscopic data for input. For a visual representation of the pressure-induced changes in elastic disorder, we then employ the obtained correlation length, the extent of non-affinity and the geometric mean of the analyzed shear modulus to construct a glass with corresponding shear modulus fluctuation in silico. On these models, we map the characteristic distribution of strain energy, which arguably reflects non-affinity in the real-world material. The observed relations are compared to short-range structural information using nuclear magnetic resonance spectroscopy as a complementary method.

## Materials and methods

### Sample material

#### Sample synthesis

The glass used in this study was prepared by conventional melt quenching from a batch of SiO_2_, MgCO_3_, CaCO_3_, and Na_2_CO_3_ to yield a glass composition of 70 SiO_2_–8 MgO–10 CaO–12 Na_2_O (mol%, confirmed by inductively-coupled plasma mass spectrometry). After grinding in a porcelain mortar, the batch mixture was melted in a platinum crucible at 1500 °C for 2 h. Subsequently, the melt was poured into a graphite mould and annealed at 530 °C for 1 h before cooling to room temperature. From the obtained glass slab, individual samples were cut to dimensions of 10 mm × 7.5 mm × 0.5 mm and polished on both faces to optical grade using CeO_2_ powder.

#### Compression method

Hot-compression was performed in accordance with established protocols^29,30^ at the ambient-pressure glass transition temperature (*T*_g_ = 533 °C) in two separate runs (1 GPa and 2 GPa), using a multizone cylindrical graphite furnace placed inside a gas pressure reactor with nitrogen as the compression medium. Details on the compression procedure and supplementary physical data of the employed glass are provided in Ref.^31^, which used the exact same glass as reference material (denoted SG0 in Ref.^31^). X-ray diffraction (Rigaku Miniflex 600) on the recovered glass samples did not reveal any evidence of crystallization following hot-compression treatment.

### Analytical methods

#### Raman scattering spectroscopy

Raman spectroscopic measurements were performed using a Renishaw inVia confocal Raman microscope equipped with a low-frequency notch filter for collecting vibrational spectra in the frequency-range of 0–200 cm^−1^. Samples were excited with an Argon ion (Ar^+^) laser emitting at 514.5 nm. The light was focused into the sample and collected using a confocal microscope with a 50 × objective*.* The scattered light was directed to a Rayleigh-line rejection filter to block the excitation laser light. All spectra were recorded in two polarization geometries, VV and VH, over a wavenumber region of 10–1380 cm^−1^ with 2 cm^−1^ resolution (using a diffraction grating with 2400 lines/mm). The polarization was controlled by means of a polarizer/half-wave plate set-up inserted in the laser beamline between the notch filter and the monochromator.

#### Nuclear magnetic resonance spectroscopy

^29^Si NMR MAS spectra were collected on a Q-OneTec 500 MHz spectrometer operating at 11.7 T (99.3 MHz for Si) equipped with a 7 mm rotor and spinning at 5 kHz. For this, samples were crushed manually using a mortar and pestle. Relaxation delays of 600 s were required for complete relaxation of a 90° pulse (8 μs), allowing for only 128 free-induction decays to be collected per sample. Chemical shifts were referenced to tetramethylsilane (TMS) and the spectrometer field was shimmed directly before data collection to obtain a sharp reference peak (38 Hz). Care was taken to collect a stator background spectrum with the same experimental conditions and subtract it from all samples. Due to the expected small differences between spectra, only a small exponential line-broadening (100 Hz) was applied during processing in TopSpin. After phasing in TopSpin, the spectra, including spinning sidebands, were deconvoluted in DmFit^32^ with all parameters being varied except for the spinning speed, which was locked at 5 kHz. The Monte Carlo error of the model was calculated for 600 replicates; the standard deviation of the replicates was used to determine the error for two standard deviation intervals (equal to the 95% confidence level). Additionally, the error from the peak width of the reference compound, TMS, was also added to the error of the peak positions and widths.

#### Ultrasonic echography

An echometer (Karl Deutsch GmbH & Co. KG) with a piezoelectric transducer operating in the frequency range of 8–12 MHz was employed to determine longitudinal and transversal sound wave propagation times, from which the transversal and longitudinal sound velocities ($\mathrm{v}$_*T*_ and $\mathrm{v}$_*L*_) were deduced, and shear (${G}_{\mathrm{exp}}$), bulk (${K}_{\mathrm{exp}}$) and Young’s (${E}_{\mathrm{exp}}$) moduli and Poisson’s ratio $\upnu$ were calculated.

Densities $\rho$ were taken from Ref.^31^. The atomic packing density ${C}_{\mathrm{g}}$, was estimated according to the MM model^5^ (Table 1).

**Table 1:** Physical and macroscopic (experimental) mechanical properties of investigated glasses: density $\rho$, longitudinal and transversal sound wave velocity (${v}_{\mathrm{L}}$ and ${v}_{\mathrm{T}}$), shear modulus ${G}_{\mathrm{exp}}$, bulk modulus ${K}_{\mathrm{exp}}$, Young’s modulus ${E}_{\mathrm{exp}}$, Poisson’s ratio *ν and* calculated atomic packing density ${C}_{\mathrm{g}}$.

SLMS glass samples	Density^31^ (g/cm^3^)	${v}_{\mathrm{L}}$ (m/s)	${v}_{\mathrm{T}}$ (m/s)	${G}_{\mathrm{exp}}$ (GPa)	${K}_{\mathrm{exp}}$ (GPa)	${E}_{\mathrm{exp}}$ (GPa)	$\upnu$	${C}_{\mathrm{g}}$
pristine	2.563 ± 0.002	5703 ± 10	3408 ± 4	29.8 ± 0.1	43.7 ± 0.4	72.8 ± 0.7	0.222 ± 0.003	0.508
1 GPa	2.630 ± 0.002	5845 ± 10	3477 ± 4	31.8 ± 0.1	47.5 ± 0.5	78.0 ± 0.8	0.226 ± 0.003	0.521
2 GPa	2.683 ± 0.002	5944 ± 10	3534 ± 4	33.5 ± 0.1	50.1 ± 0.5	82.2 ± 0.8	0.227 ± 0.003	0.532

### HET-CPA implementation

We recently reported on the implementation of HET-CPA for classifying the vibrational spectra of real-world glasses in terms of elastic disorder^28^, considering that CPA is a highly reliable spectral theory of disorder^33,34^. In the present study, we apply the same procedure to study the effect of densification while keeping the glass’ bulk chemical composition unchanged. In short, the approach requires polarized VV and depolarized VH low-frequency Raman spectra, longitudinal and transversal sound wave velocity (${v}_{\mathrm{L}}$ and ${v}_{\mathrm{T}}$), and the Debye wavenumber ${k}_{D} = \sqrt[3]{6{\pi }^{2}\rho /\overline{m} }$ with the average atomic mass $\overline{m }$ and the mass density $\rho$ for input. From these data, the vibrational density of states (VDoS) and the probability density distribution of the local shear modulus *P*(*G*) are obtained, from which the geometric mean ${G}_{0}$ and the disorder parameter ${\sigma }^{2}$ are extracted. In addition, we obtain the momentum cut-off ${k}_{e}=\sqrt[3]{2{\pi }^{2}/{V}_{c}}$, with the coarse-graining volume ${V}_{c}$ used to define the local shear moduli ${G}_{i}$. In order to justify treating ${G}_{i}$ as independent random variables, ${\xi }_{c}=\sqrt[3]{{V}_{c}}$ must be of the order of the correlation length ${\xi }_{G}$ of the local fluctuations. We make the ansatz ${k}_{e}={{A}_{\xi }/\xi }_{G}$ (with ${A}_{\xi }=11.4$). Since the fitting procedure of the experimental data to the HET-CPA equations is ill-posed, regularization is required^28^.

The non-affinity parameter $n$ is obtained from the difference between the macroscopic (experimental) shear modulus and the geometric mean, $n=1-{G}_{\mathrm{exp}}/{G}_{0}$ (where ${G}_{\mathrm{exp}}$ is always lower than ${G}_{0}$). The values of ${G}_{0}$, $n$ and ${\xi }_{G}$ are used as quantifiers of elastic heterogeneity and disorder (with $n$ being directly related to ${\sigma }^{2}$).

The obtained parameters were employed to reconstruct a two-dimensional glass with the shear modulus distribution $G\left(x,y\right)$ in silico. For this, we generated spatially correlated (2D isotropic) maps of random shear moduli which match the distribution function of *P*(*G*). In order to avoid extreme spikes in those maps, we cut 10% of the area of the *P*(*G*) from the right (where *G* approaches infinity). For spatial correlation, the random maps were convoluted with a Gaussian decay function with a standard deviation of ${\xi }_{G}/\left(2\sqrt{2}\right)$. The convolution was done by multiplication in Fourier space, followed by inverse Fourier transformation. The corresponding spatial distribution of strain energy was calculated through finite element analysis of the two-dimensional HET equation in the frequency domain, with an additional harmonic stimulation on one of the four boundaries of the isotropic “2D glasses”, and spatially invariant Poisson’s ratio and mass density. We initially identified about 200 eigenmodes around the VDoS Boson peak position, and subsequently calculated the frequency response (total displacement field, total strain energy) at a frequency chosen near the resonance (the response spectra for the two examples shown in Fig. 4 are provided in Fig. S4). Thereby, *strain energy* denotes the total elastic energy density ${U}_{s}$ (J/m^3^) at pixel resolution in 2D, derived from the stiffness tensor ***C*** and the elastic strain tensor ${{\varvec{\varepsilon}}}_{elastic}$ under the assumptions of linear elasticity and zero initial stress. Since the simulation is in the frequency domain, values of ${U}_{s}$ are time-averaged over one period.

## Results and discussion

### Structural characterization

Overview spectra of polarized VV and depolarized VH Raman scattering are shown in Fig. S1a,b, respectively, for the pristine and the hot-compressed SLMS glasses. For frequencies > 200 cm^−1^, the Raman scattering bands of soda-lime silicate glasses are well documented in literature^35–38^, and also the effect of hot-compression to 2 GPa was reported previously^31^. In short, hot-compression at such comparably low pressure does not lead to significant changes in the intermediate-frequency region of the Raman spectra, in particular, in the depolarized case. Furthermore (other than in cold-compression experiment), the liquid fully relaxes into the compressed state while pressure is applied; after cooling and decompression at room temperature, the applied pressure corresponds to the *fictive pressure p*_f_ of the obtained glass. The only notable variation in the Raman scattering is in the main band ΔL_1/2_ (the half width at half maximum) of the VV spectrum, which exhibits a slight upshift, increases in intensity and becomes somewhat sharper with increasing compaction (Fig. S1c,d). These observations indicate a slight overall increase in phonon energy due to decreasing average atomic distance and a sharper distribution of the Si–O-Si angles^39–41^.

**Figure 1:**
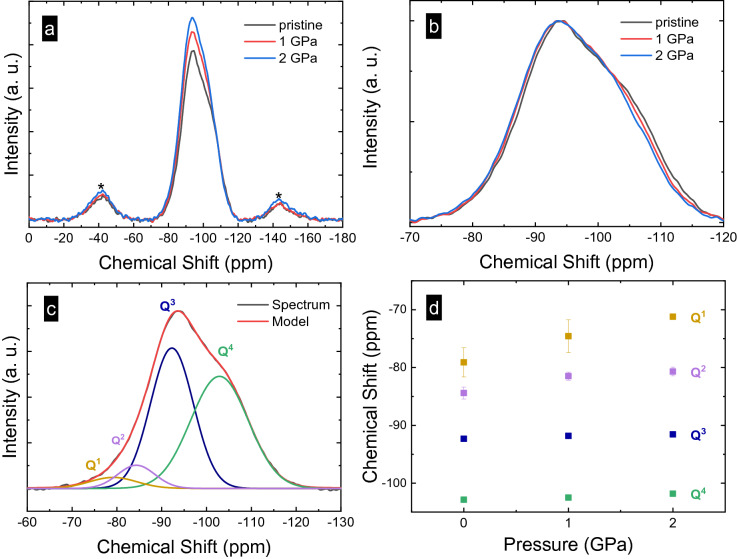
(**a**) ^29^Si MAS NMR spectra of SLMS glasses taken from pristine (0.1 MPa) and hot-compressed samples (1 GPa and 2 GPa); (**b**) Close-up of intensity normalized ^29^Si MAS NMR spectra; (**c**) Deconvolution of spectrum for the pristine glass; (**d**) Chemical shift parameters of SLMS glasses as a function of pressure during hot-compression.

To further observe the structural changes caused by hot-compression, we turned to ^29^Si MAS NMR spectroscopy. In Fig. 1a, a clear trend is seen in the evolution of the ^29^Si central peak as a function of applied pressure. This indicates that the pressure effect is preserved after crushing the sample for NMR analyses. On closer inspection, there is a consistent increase in chemical shift (indicating more shielded nuclei) with increasing compaction (Fig. 1b). Peak deconvolution (shown by way of example for the pristine glass sample in Fig. 1c, and for the compacted samples in Fig. S2) indicates dominance of mostly Q^3^ and Q^4^ structural units, with a minor amount of Q^2^ and Q^1^. This is to be expected based on the modifier-to-silica ratio of the SLMS glass. Although NMR studies of similar glasses (in particular, soda-lime silicate without MgO^42,43^) used only two peaks (Q^3^ and Q^4^ units) to fit the ^29^Si band for this ratio of glass former to glass modifier, we found that four peaks were needed to reproduce the central peak adequately. This is possibly due to our base glass composition having larger amounts of MgO (possibly producing four-coordinated Mg^2+^ sites that may require charge compensation) and CaO (rather than Na_2_O), since alkaline earth ions are known to increase the disproportionation reaction and lead to a larger spread of Q^n^ units^44^. In fact, it was argued that alkaline earth silicates are consistently underfit, and that five peaks (Q^0^–Q^4^) should be used. Nonetheless, fitting the spectra with more than four Q^n^-unit peaks was not necessary in our case. The fitting of the spinning sidebands, in addition to the central peak, resulted in very reliable fits, as can be seen in the small error (see Table 2).

**Table 2:** Deconvolution parameters for ^29^Si MAS NMR spectra of SLMS glasses obtained for pristine (ambient) and hot-compressed (1 GPa and 2 GPa) samples.

^29^Si MAS NMR fit parameters		Samples		
		Pristine	1 GPa	2 GPa
Chemical shift (ppm)	Q^1^	− 79 ± 3	− 75 ± 3	− 71.2 ± 0.5
	Q^2^	− 84 ± 1	− 81.5 ± 0.7	− 80.7 ± 0.7
	Q^3^	− 92.3 ± 0.2	− 91.8 ± 0.09	− 91.6 ± 0.1
	Q^4^	− 102.9 ± 0.2	− 102.5 ± 0.3	− 101.8 ± 0.3
Peak width (ppm)	Q^1^	12 ± 2	7 ± 3	3 ± 1
	Q^2^	10 ± 1	9 ± 2	10.6 ± 0.9
	Q^3^	11.2 ± 0.4	12.5 ± 0.4	12.5 ± 0.3
	Q^4^	15.2 ± 0.2	14.6 ± 0.2	14.8 ± 0.2
Relative area (%)	Q^1^	4 ± 2	1 ± 1	0.11 ± 0.06
	Q^2^	7 ± 4	4 ± 2	5 ± 1
	Q^3^	43 ± 4	51 ± 3	48 ± 3
	Q^4^	47 ± 1	44 ± 2	45 ± 2
(°, Q^4^ only)		135.3	134.7	133.5
Si–Si (Å)		3.034	3.027	3.014
%-volume reduction		–	− 0.69	− 1.95
[NBO]/[O]		0.29 ± 0.03	0.27 ± 0.02	0.26 ± 0.02
[NBO]/[Si]		0.67 ± 0.07	0.61 ± 0.04	0.59 ± 0.04

Chemical shifts of Q^n^ units, peak width, relative abundance (%) of Q^n^ species, calculated average ∠Si–O–Si bond angle, Si–Si distance, volume reduction and average number of non-bridging oxygen per silicon atom [NBO]/[Si] and oxygen atom [NBO]/[O].

The chemical shift values (Fig. 1d and Table 2) are in-line with observations on other soda-lime silicates^42,45^, although the present results are generally on the low side of the reported ppm range, likely due to the presence of magnesium. Indeed, this is especially true for our Q^4^ chemical shift value (~ − 103 ppm), whereas pure crystalline SiO_2_ compounds have their Q^4^ peaks located <  − 107 ppm (and have a mean Si–O–Si angle of > 143.2°^45,46^). When we use equations relating chemical shift to average ∠Si–O–Si bond angle for Q^4^ units only (from either Malfait et al*.*^46^ or Oestrike et al*.*^47^), we calculate an apparent average value of 135.3° for the Q^4^ units in our pristine sample (Table 2). This is much lower than the mean ∠Si–O–Si of 149° of compositionally similar soda-lime glasses reported by X-ray diffraction^48^ or molecular dynamics simulation studies^49,50^. There are many explanations for this difference. Modifying cations, even though not directly connected to Q^4^-units can have large influences on the NMR peak position^51,52^, with MgO known to cause a more deshielded Si nucleus (higher ppm)^53^. Although insufficient relaxation times have also been known to affect ^29^Si chemical shifts^46^, short delays result in higher ppm, not lower ppm as we observe here.

Next, we note that in a soda-lime silicate glasses, a Si–O–Si bond necessarily involves another SiO_4_^4−^ tetrahedron, therefore, some Q^4^-units are necessarily connected to Q^2^- and Q^3^-units. Given that Q^2^ units in diopside and α-CaSiO_3_ have ∠Si–O–Si of 135.93° and 135°^54,55^, respectively, and Q^3^ units in crystalline Li_2_Si_2_O_5_ and α-Na_2_Si_2_O_5_ have ∠Si–O–Si of 128° and 138.93° in the presence of a cation^56–58^, respectively, an average Q^4^ Si–O–Si angle of 135.3° appears to be physically reasonable for a modified silicate composition^57^. This is further corroborated by our calculated Si–Si distance for the ambient pressure sample, 3.043 Å (assuming constant Shannon-Prewitt ionic radii^59^, although the Si–O bond has been found to lengthen with pressure, it is a much smaller effect, only 0.02 Å reduction^60^), which is in agreement with the both the X-ray diffraction data (3.14 Å)^48^ and observations on silicate crystals with similar composition (3.0–3.1 Å)^61^.

Rather than further discussing the absolute value of the bond angle, in the following, we focus on the relative effect of densification, *i.e.*, the narrowing of the Si–O–Si bond-angle of Q^4^ units only (not Q^2^ and Q^3^), simply because it is the most well-studied and, therefore, potentially reliable trend related to the ^29^Si chemical shift. Indeed, Mackenzie and Smith^62^ caution against combining chemical shift and ∠Si–O–Si data into a single relationship, as the chemical shift is greatly affected by next-nearest neighbors; therefore, it can strictly be applied only to alike materials. It is much more challenging to understand the structural changes in chemical shift for the Q^2^ and Q^3^ units, partly because there are no model systems with our exact glass composition, but also because of the action of the modifying cation to pressure. For diopside crystals (MgCaSi_2_O_6_, all Q^2^) under pressure, it was found that the volume of the modifier cation coordination sphere compresses much more than that of the SiO_4_^4−^ tetrahedron, 2.5% vs. < 1%, respectively, for pressures between ambient and 2.36 GPa; in fact Levien and Prewitt^63^ found that the compression of diopside is controlled by the ‘directions and compressibilities of the bonds in the cation polyhedra and not by the chains of the silicate tetrahedra’—thus, the exact way in which densification manifests itself on short-range order in the present glasses remains unclear, especially regarding Q^2^ and Q^3^-units and their neighbouring cations.

Using the reduction of the Si–Si distance, which is caused by the narrowing of the Si–O–Si angle in Q^4^ units, we find a corresponding volume reduction of 0.69% and 1.95% for hot-compression at 1 and 2 GPa, respectively. This compares to the respective macroscopic densification of 2.61% and 4.66% observed in mass density^31^. The difference arguably reflects the preferential compaction of ‘soft’ cation sites^64^, which are not fully reflected in the Si–O–Si angle when only Q^4^ units are considered in the analysis of densification (as the relation between the chemical shift of Q^2^ or Q^3^ and the involved Si–O–Si bond angle is unknown^62^). Again, we are unsure about the exact structural response of the Q^2^- and Q^3^-units upon densification, except that their chemical shifts generally increase under pressure, too (in ppm), indicating a narrowing of the average ∠Si–O–Si. However, the magnitude of the narrowing is unknown.

In a more in-depth ^17^O NMR study of pure SiO_2_ crystals and glasses^60^, pressures of 8 and 13.5 GPa, respectively, were found to decrease the Si–O–Si angle significantly (with an increase in mean Si–O bond length). This study also noted that there may be a hard limit to the sharpest energetically favourable ∠Si–O–Si, 130°, and rather than all angles necessarily decreasing equally, sharper ∠Si–O–Si simply became more prevalent (which decreased the *average* ∠Si–O–Si). The width of the bond-angle and bond-distance distributions did not change significantly, however, the shape of the ∠Si–O–Si distribution changed, becoming more Lorentzian in shape (where even extreme ∠Si–O–Si values have significant population). This is an example of a structural preference affecting the shape of the probability distribution, which could lead to ill-fitted parameters if not taken into consideration.

The fraction of different Q^n^ species corresponds to what has been reported elsewhere^49^ in the case of a similar manganese-containing soda-lime silicate glass (72.2 SiO_2_–5.5 MgO–8.9 CaO–12.3 Na_2_O–1.1 Al_2_O_3_ (mol%)). In particular, the Q^4^ species population was found to be higher than that of Q^3^, Q^2^ or Q^1^, which supports the relevance of our deconvolution. The [NBO]/[O]_NMR_ ratio calculated from NMR, 0.238, is somewhat lower than that expected from composition, 0.34, and that from a soda-lime silicate glass of similar composition without MgO, 0.29^42^; we believe that the presence of Mg^2+^ could be the reason for this observation^65^. The small decrease in [NBO]/[O] in Table 2 is concomitant with an increase in ‘free oxide’ (FO) according to 2 NBO → 1 BO + FO, which may confirm that under compression, there is preferential compaction of soft ion sites. More importantly, we do not expect a large change in [NBO]/[O] at the relatively low pressures we applied here (Table 2).

### Elastic heterogeneity and compaction-induced homogenization

**Figure 2:**
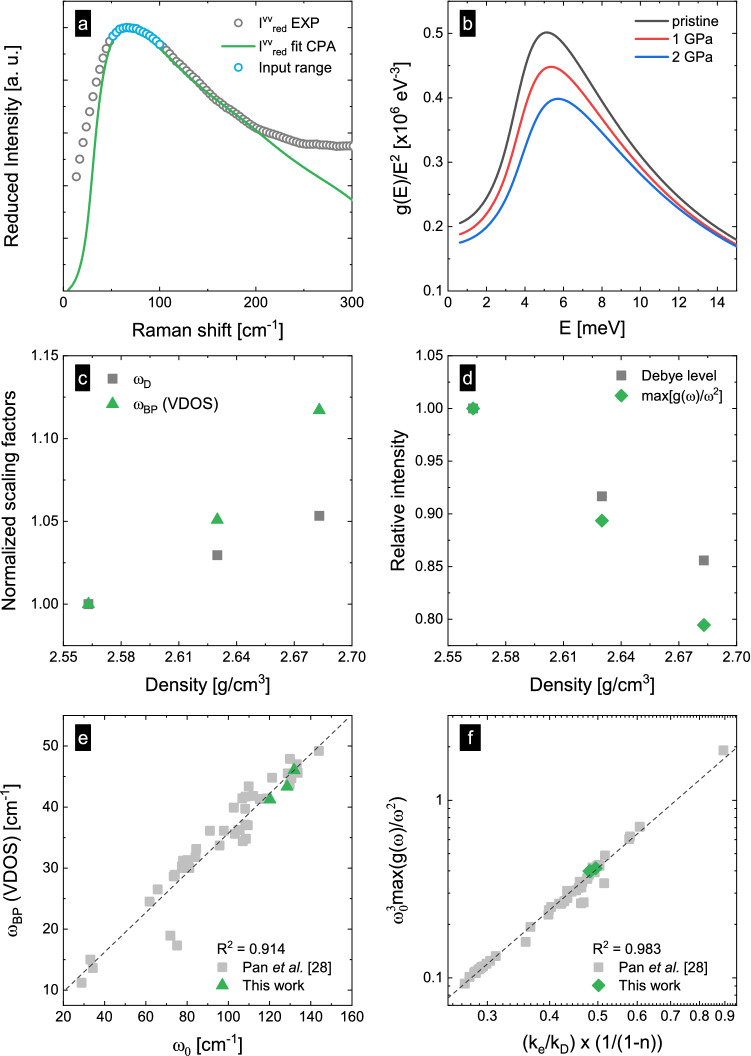
(**a**) Low-frequency Raman spectrum (reduced intensity ${I}_{\mathrm{red}}^{\mathrm{vv}}$) of the pristine SLMS glass (grey scattered points) and HET-CPA fit (green solid line). The blue circles mark the input range of the fit (50–100 cm^−1^). (**b**) Reduced vibrational density of states g(*E*)/*E*^2^ obtained from HET-CPA for the pristine and the hot-compressed glasses. (**c**) Variation of the scaling factors relative to the Boson peak frequency ${\upomega }_{\mathrm{BP}}$ (VDOS) and to the Debye frequency ${\upomega }_{\mathrm{D}}$ as functions of the glass’ mass density. (**d**) Variation of the relative intensity the Boson peak intensity ${I}_{BP}$ (VDOS) and the Debye level as functions of the mass density. (**e**) Boson peak frequency ${\upomega }_{\mathrm{BP}}$ (VDOS) as a function of ${\upomega }_{0}$. (**f**) Double-logarithmic plot of the maximum of ${\upomega }_{0}^{3}\mathrm{max}[\mathrm{g}(\upomega )/{\upomega }^{2}]$ vs. $\left({k}_{e}/{k}_{d}\right)\times \left(1/1-n\right)$. The grey dashed lines in panels (**e**,**f**) are linear data fits (with slope of 0.33 in **(e)** and 2.42 in **(f)**).

An example of fitting low-frequency Raman data using HET-CPA is provided in Fig. 2a for the pristine glass sample. The spectrum is displayed in reduced scale, ${I}_{\mathrm{red}}\left(\omega ,T\right)={I}_{\mathrm{n}}\left(\omega ,T\right)/[m\left(\omega ,T\right)+1]$^27^. The function *n* $(\omega ,T)$ is the Bose–Einstein distribution function for the phonon occupation at frequency $\omega$ and temperature *T*. The HET-CPA data fit reproduces the spectral shape of the reduced low-frequency Raman spectrum (< 200 cm^−1^), with a standard error regression error of *S* = 0.071 (in the range 30–200 cm^−1^). All output parameters are listed in Table 3. We note that HET underestimates the experimental Raman scattering intensity for $\omega \to 0$, as $I\left(\upomega \right)\propto {\upomega }^{2}$^66^. This is related to quasi-elastic scattering (QES)^67^, which is not considered in HET.

**Table 3:** Model output parameters: Boson peak position *ω*_BP_ (VDoS), geometric mean shear modulus ${G}_{0}$, momentum cut-off ${k}_{e}$, coarse-graining length ${\xi }_{\text{c}}$, correlation length ${\xi }_{G},$ disorder parameter ${\sigma }^{2}$ and non-affinity $n$.

SLMS glass samples	*ω*_BP_ (cm^−1^)	${G}_{0}$ (GPa)	${k}_{e}$ $({\mathrm{\AA }}^{-1})$	${\xi }_{\text{c}}$(nm)	${\xi }_{G}$(nm)	${\sigma }^{2}$	${\text{n}}$
Pristine	41.23 ± 0.79	39.57 ± 0.33	0.594 ± 0.013	0.455 ± 0.010	1.917 ± 0.042	0.985 ± 0.032	0.247 ± 0.006
1 GPa	43.33 ± 0.34	41.89 ± 0.22	0.607 ± 0.005	0.445 ± 0.04	1.875 ± 0.016	0.957 ± 0.020	0.241 ± 0.004
2 GPa	46.06 ± 0.85	43.77 ± 0.41	0.632 ± 0.019	0.428 ± 0.013	1.803 ± 0.050	0.924 ± 0.036	0.234 ± 0.007

Figure 2b depicts the reduced VDOS from HET-CPA for pristine and hot-compressed glasses. From these spectra, the Boson peak frequency ${\upomega }_{\mathrm{BP}}$ (VDoS) is extracted as the frequency at which g(ω)/ω^2^ reaches its maximum. It shifts toward higher frequencies and decreases in intensity ${I}_{\mathrm{BP}}$ = max[g(ω)/ω^2^] with hot-compression at increasing pressure. Similar observations have already been made in semi-empirical studies of hot- or cold-compressed glasses (mostly using empirical coupling factors for obtaining VDoS from Raman scattering data)^68–70^. The up-shift of ${\upomega }_{\mathrm{BP}}$ is usually related to a qualitative decrease in the length scale of heterogeneity^71,72^, and the decrease in intensity indicates a decrease in the number of soft modes. Evaluation by HET now provides a quantitative and purely physical basis for this conjecture^28,73,74^.

Comparing the vibrational properties of different solids requires prior scaling (normalization) with the continuous medium properties expressed in terms of the Debye frequency ${\upomega }_{D}$ and the Debye level ${\mathrm{A}}_{D}$ (${\mathrm{g}}_{D}\left(\upomega \right)={\mathrm{A}}_{D}{\upomega }^{2}$)^75^. We now verify whether the shift of ${\upomega }_{\mathrm{BP}}$(VDoS) and the decrease of its amplitude ${I}_{\mathrm{BP}}$ (or max[g(ω)/ω^2^]), observed in Fig. 2b represent a deviation from continuous medium transformation, that is, non-affinity. For this, we compare first the density dependence of ${\upomega }_{\mathrm{BP}}$ and that of ${\upomega }_{D}$ (with ${\upomega }_{D}\approx {\left(9{\pi }^{2}\rho \right)}^{1/3}\sqrt{{G}_{exp}/\rho }$)^76^ as reported in Fig. 2c. Furthermore, the variation of the Boson peak amplitude ${I}_{\mathrm{BP}}$ (max[g(ω)/ω^2^]) is compared to the variation of the Debye level ${\mathrm{A}}_{D}$ (with ${\mathrm{A}}_{D}=3 {{\upomega }_{D}}^{-3}\approx {\left[3{\pi }^{2}\rho {\left({G}_{exp}/\rho \right)}^{-3/2}\right]}^{-1})$^76^ as shown in Fig. 2d. Both datasets in Fig. 2c,d are normalized to a reference value (we use the mass density at room temperature). In Fig. 2c, we observe that the frequency shifts induced by hot-compression are about one order of magnitude higher than those of the Debye frequency. A similar behavior is observed in Fig. 2d, where changes of the Boson peak intensity systematically exceed the changes in the Debye level. These findings are consistent previous studies of hot and cold-compressed glasses and polymers^76–78^ which clearly demonstrate that the glass does not behave as an isotropic, homogeneous elastic continuum, at least upon compression. As the continuum medium properties (${\upomega }_{D}$ and ${\mathrm{A}}_{D}$) are mostly determined from the macroscopic shear modulus ${G}_{exp}$, we deduce that non-affine compression (preferential compression of soft modes) affects not only the macroscopic shear modulus ${G}_{exp}$ (+ 12.5%), but also its spatial fluctuation^76^. During the last decade, various studies have been carried out with the aim to explain the non-Debye scaling (the failure of scaling observed in Fig. 2c,d) during densification^68,78,79^, variations in cooling rates^80,81^ or adaptions of chemical composition^82^. Examining a broad range of glass compositions, two phenomenological correlations were recently discovered which link vibrational disorder and boson peak parameters to fluctuating elasticity^28^. First, a one-to-one correlation was found between the characteristic frequency ${\upomega }_{0}={k}_{e}\sqrt{{G}_{0}/\rho }$ and the VDoS Boson peak position, ${\upomega }_{\mathrm{BP}}=0.333 {\upomega }_{0}$. Secondly, a power-law relationship was revealed for the Boson peak intensity, ${\upomega }_{0}^{3}\mathrm{max}[\mathrm{g}(\upomega )/{\upomega }^{2}]=2.19 {\left(\left({k}_{e}/{k}_{d}\right)\times \left(1/1-n\right)\right)}^{2.42}$. Both of these relations exhibited applicability to the broadest range of glass chemistries. Interestingly, this also appears to hold when the chemical composition is kept unchanged, but the fictive pressure (and thus, glass density) is varied: in Fig. 2e,f, we show that the present compacted SLMS glasses also fall perfectly into the reported trend (it remains to be explored how this will hold for other glass compositions, or when the observed range of pressures is extended).

**Figure 3:**
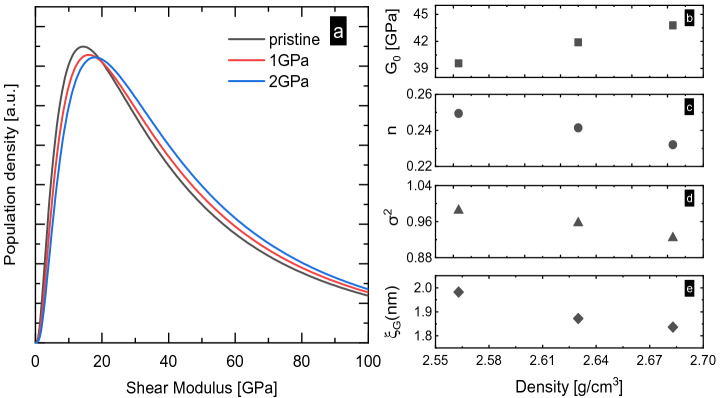
(**a**) Population density distributions of local shear modulus *P*(*G*) of SLMS glasses in their pristine state and after hot-compression (1 GPa and 2 GPa). (**b**) Typical shear modulus (geometric mean) ${G}_{0}$, (**c**) non-affinity parameter *n*, (**d**) disorder parameter ${\sigma }^{2}$ and (**e**) characteristic length ${\xi }_{G}$ as functions of mass density.

The population density distributions of the shear modulus of pristine and hot-compressed SLMS glasses are presented in Fig. 3a. Our choice of a log-normal distribution was initially motivated by the assumption that the fluctuation in shear modulus (in the glassy state) is related to liquid dynamics (in the melt from which the glass was originally derived). The validity of this approach is confirmed by Köhler et al*.*^83^, who emphasized the suitability of log-normal distributions in covering sufficiently high degrees disorder to represent real-world glasses (over uniform or truncated Gaussian functions).

As shown in Fig. 3b, the typical shear modulus ${G}_{0}$ (the geometric mean of the log-normal distribution) increases linearly with densification. This is intuitively expected, because also the macroscopic shear modulus ${G}_{\mathrm{exp}}$ increases (Table 1). However, on closer inspection, we find that *G*_0_ seems to be less affected by densification as compared to the macroscopic shear modulus, with an increase of 10.6% versus ~ 12.4% observed after hot-compression at 2 GPa. This is a direct manifestation of the deviation from the continuum medium transformation (CMT) model; it indicates non-affine compaction, whereby we may assume that ‘soft’ regions compact preferentially over more rigid regions, in accordance with our previous discussion of the variations in Si–O-Si angle observed by NMR.

Several explanations have been proposed for the variation of the macroscopic shear modulus ${G}_{\mathrm{exp}}$ with densification in different types of glasses, typically referring to the change of packing density or to variations in network topology (such as changes on the Si–O–Si angle and the distribution of ring sizes, the formation of highly-coordinated defect states or increasing coordination numbers on network-forming species). In our case, we obtain an increase in ${C}_{\mathrm{g}}$ by 4.72% for hot-compression at 2 GPa, see Table 1. This represents relatively mild compression, for which we do not expect significant variations on the Si coordination number^84^ (in agreement with our NMR and Raman analyses).

Besides *G*_0_, the *P*(*G*) is characterized by a second parameter, the disorder parameter ${\sigma }^{2}$. As a result of hot-compression at 2 GPa, we observe a decrease in ${\sigma }^{2}$ by ~ 7% (noteworthy, ${\sigma }^{2}$ being a quadratic function in exponential space, Fig. 3d). This manifests in a significantly sharper *P*(*G*), whereby the sharpening occurs through the reduction of ‘soft modes’: the high-modulus boundary of *P*(*G*) is almost unaffected by hot-compression, whereas the shift occurs on the lower edge. This relative decrease in the number of ‘soft modes’ causes a decrease in the difference between the macroscopic modulus *G*_exp_ and the geometric mean *G*_0_^28^ and, thus, a decrease in the non-affinity parameter *n* (in the present case, by about 5.5% following hot-compression at 2 GPa, Fig. 3c). This result is in agreement with that of Pan et al.^28^; as shown in Fig. S3, the non-affine reduction of the macroscopic shear modulus *G*_exp_ occurs in a wide range of glasses.

With the variations in *P*(*G*), the correlation length ${\xi }_{G}$ decreases by ~ 6% after hot-compression at 2 GPa (Fig. 3e). This compares to a simultaneous change in packing density by ~  + 4.7% (Table 1) and again reflects the deviation from the CMT approximation, where the effect of compression on ${\upomega }_{\mathrm{BP}}$ is two to three times as high as the one on ${\upomega }_{\mathrm{D}}$ (inset of Fig. 2c). It is interesting to note that this roughly matches the amount by which the variation of ${\xi }_{G}$ exceeds the expectation derived from the (macroscopic) packing density alone. The ‘excess’ of ${\xi }_{G}$ variation appears to be related to the decrease in non-affinity; both parameters affect the position of the boson peak^28^ and, therefore, the deviation from CMT.

**Figure 4:**
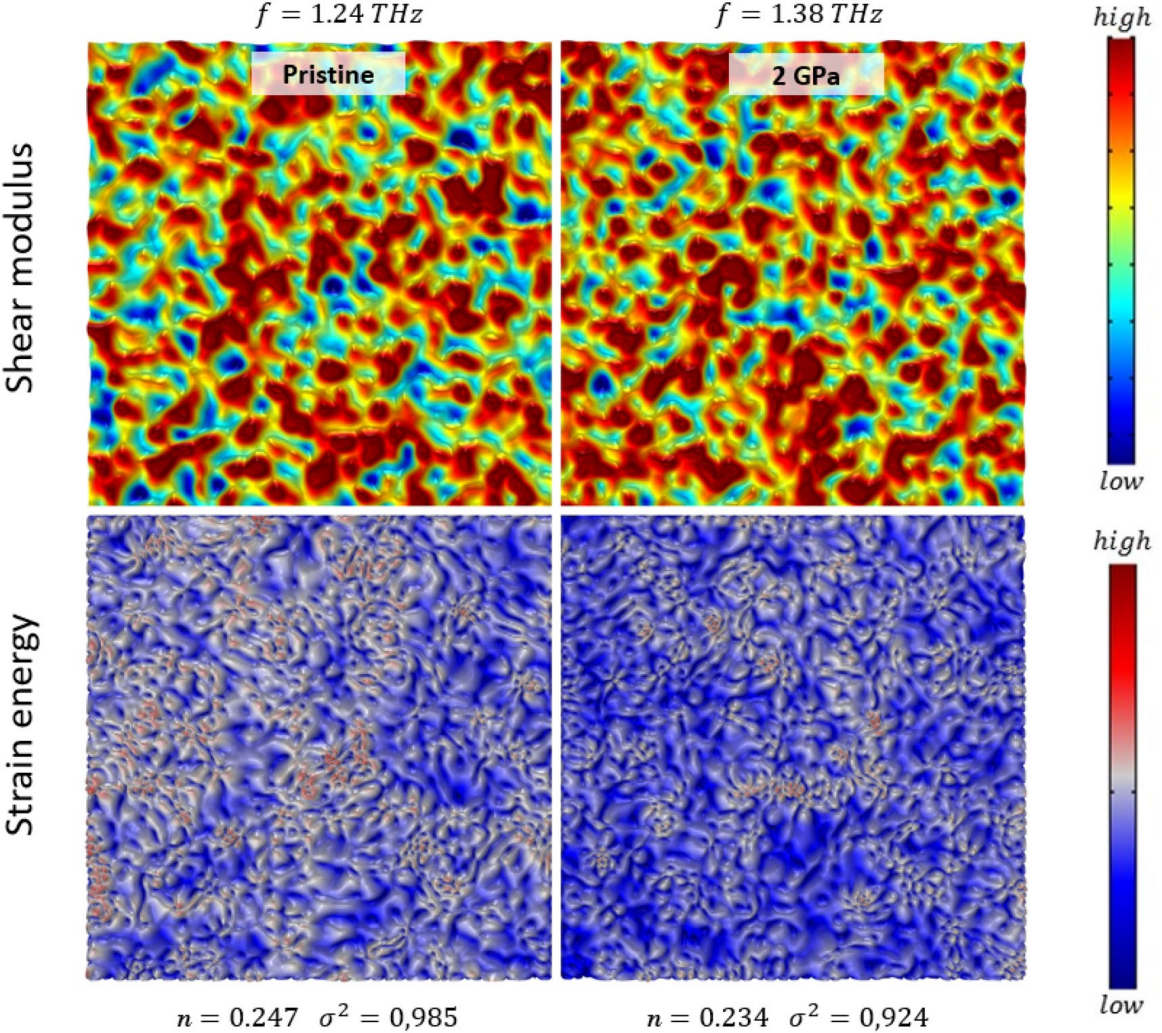
(top) Real-space two-dimensional reconstruction of the local elastic heterogeneity *G*(x,y) as obtained for pristine and hot-compressed (2 GPa) SLMS glass by HET-CPA. The image size is 50 nm × 50 nm. The top-label indicates the resonance frequency used for excitation in the strain energy maps (bottom). The corresponding non-affinity parameter *n* and the disorder parameter *σ*^2^ are indicated below the strain energy panels.

Two-dimensional in silico reconstructions of the HET-CPA parameters of the pristine and compressed SLMS glasses are shown in Fig. 4. They act as two-dimensional representations of *P*(*G*), assuming random spatial fluctuation with an autocorrelation length of ${\xi }_{G}$. Therefore, while all physical information is strictly contained within the parametric descriptors, the reconstructions provide visual access for further interpretation of the effect of hot-compression. As the primary features, both the decreasing length scale and the decreasing non-affinity are reflected in these maps, with the microstructure becoming more fine-grained as a result of hot-compression. The strain energy maps reflect the response of a material with the constructed *G*(*x,y*) (that is, for disorder statics reflecting those of the present SLMS glasses) to acoustic stimulation. Clearly, the strain energy becomes more homogeneously distributed as a result of compaction, with softer regions (low *G*) decreasing in size and less-intense local spikes in stored energy, thus, more homogeneous energy distribution (whereby sound transport occurs preferentially across the percolating hard regions: the strain energy maps provide a clear rationale for the increase in macroscopic elastic moduli as a result of compaction, which exceeds the CMT prediction).

Possible relations between structural heterogeneity, glass densification and variations on short and intermediate range order have been discussed on various occasions^85–87^, often making use of the ‘non-continuous structure model’^72,88^. This model conjectures a relation between ${\upomega }_{BP}$ and the characteristic size and shape of rigid, dispersed objects in a model morphology of ‘cohesive’ and ‘soft’ nanodomains. The size of the characteristic length scale proposed by the non-continuous structure model is on the same order of magnitude as the one found through HET in silico^19^ or HET supplied with experimental data for the broadest range of glass chemistries^28^, however, the descriptors obtained through HET-CPA have clear physical meanings. For example, instead of an empirical shape factor for dispersed nano-objects, we obtain direct access to non-affinity and a more realistic morphology such as depicted in Fig. 4. An interesting explanation for elastic homogenization following structural compaction beyond CMT was brought forth by Leonforte^89^. Using mode-projection analysis, it was shown for some glass formers (SiO_2_^90,91^, P_2_O_5_^92^) that the ‘boson peak’ is related to rotational (F_1_) modes of network-forming tetrahedra. The tetrahedra may rotate in directions opposite or similar to their neighbours. In such cases, elastic homogenization following densification can be related to the competition between connected tetrahedral units with similar and with opposing rotation. Increasing the pressure constrains the number of opposing inter-tetrahedral rotations, thereby reducing inhomogeneous particle rearrangements^89^.

## Conclusions

In summary, we studied the effect of structural compaction on the statistics of elastic disorder in a soda lime magnesia silicate glass. HET-CPA was used with THz Raman spectroscopic data to extract variations in the log-normal distribution of the local shear modulus for densification by up to 5%. These variations were found to occur primarily on the soft end of the distribution, as also reflected in a decrease in the non-affine part of the shear modulus; compaction occurred preferentially in soft modes, which lead to overall homogenization of the spatial strain energy distribution. The pressure-induced increase in macroscopic elastic moduli is attributed to this homogenization effect (which exceeds the continuous medium approximation). Within the considered pressure range, hot-compression of SLMS glasses reveals the same relationship between the boson peak position and the (local) characteristic frequency as was previously reported for the broadest variability of glass chemical compositions. It remains to be explored how experiments at higher pressure and/or the use of glasses with variable pressure dependence of (short-range) structural motifs would further affect this picture.

## Supplementary Information

Supplementary Information.

## Data Availability

The data that support the findings of this study are shown in the manuscript or supporting information, or available from the corresponding author on reasonable request.
