# Supplementary material for: Vibrational disorder and densification-induced homogenization of local elasticity in silicate glasses
Source: Sci Rep. 2021 Dec 27;11:24454. doi: 10.1038/s41598-021-04045-6 (PMC8712522; doi:10.1038/s41598-021-04045-6)
Supplement: Supplementary file 1 — Supplementary Information. [file 41598_2021_4045_MOESM1_ESM.docx]

**Vibrational disorder and densification-induced homogenization of local elasticity in silicate glasses**

Omar Benzine^1^, Zhiwen Pan^1^, Courtney Calahoo^1^, Michal Bockowski^2^, Morten M. Smedskjaer^3^, Walter Schirmacher^4^, Lothar Wondraczek^1*^

*^1^ Otto Schott Institute of Materials Research, University of Jena, 07743 Jena, Germany*

*^2^ Institute of High-Pressure Physics, Polish Academy of Sciences, 01-142 Warsaw, Poland*

*^3^ Department of Chemistry and Bioscience, Aalborg University, 9220 Aalborg, Denmark*

*^4^Institute of Physics, University of Mainz, 55099 Mainz, Germany*

* Corresponding author.

Tel.: +49(0)3641 948500

E-mail address: [*lothar.wondraczek@uni-jena.de*](mailto:lothar.wondraczek@uni-jena.de) (L. Wondraczek)

**Supplementary Information Supplementary Information**

Raman scattering spectra collected in VV and VH geometry on pristine and compacted glasses, and deconvoluted ^29^Si MAS NMR spectra of compacted glasses are available in Figure S1 and S2, respectively. Figure S3 shows the relation between non-affinity and disorder parameter for a wide range of glasses. Figure S4 shows the frequency response of constructed 2D glasses (see main text for details).

**Figure S1.** Polarized VV **(a)** and depolarized VH (**b**) Raman spectra of soda-lime silicate glasses in their pristine state (0.1 MPa) and after hot-compression (1 GPa and 2 GPa). **(c)** Illustration of the half width at half maximum of the main Raman band ΔL_1/2_ at 495 cm^-1^ **(d)** Variation ΔL_1/2_ of as function of mass density. All spectra were normalized to the intensity of the band at around 780 cm^−1^, which is practically not affected by compression.

**Figure S2.** Deconvoluted ^29^Si MAS NMR spectra of SLMS glasses taken from hot-compressed samples (**a**: 1 GPa and **b**: 2 GPa).


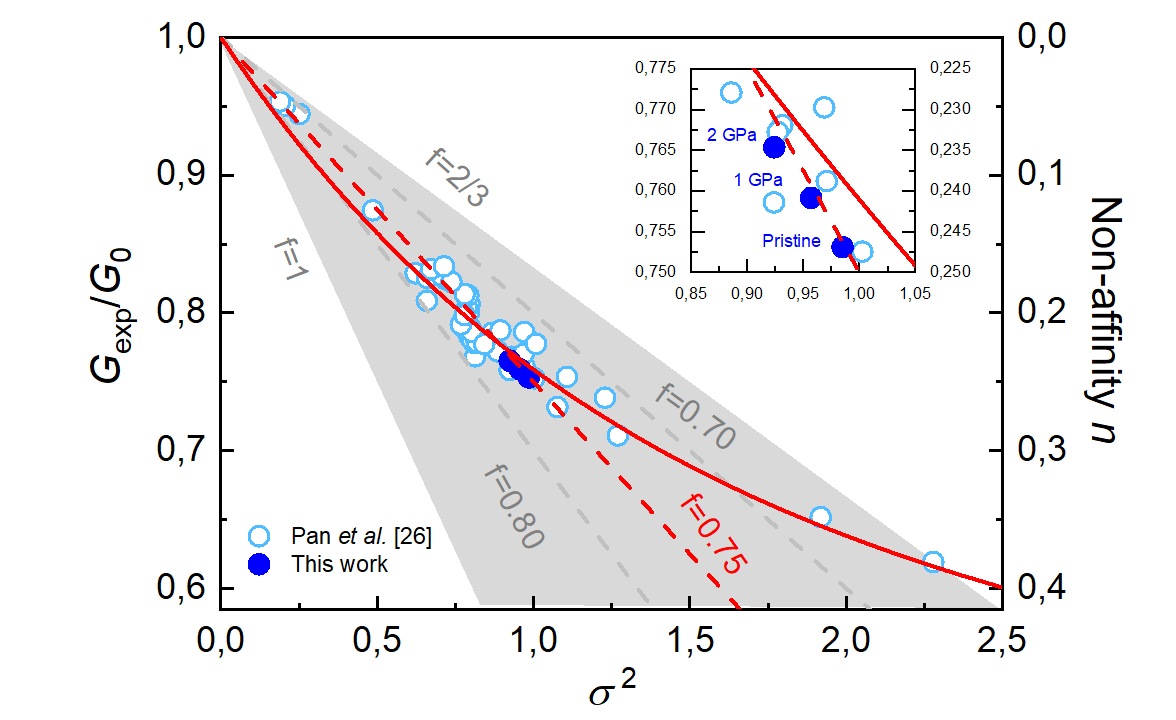


**Figure S3.** Ratio of the experimental shear modulus $G_{\exp}$ and the typical shear modulus $G_{0}$ vs the disorder parameter $\sigma^{2}$ for a wide range of glassy materials. A close-up highlighting the glasses investigated in the present study is shown in the inset. The function $f\left( k \right)$ (the curved red line, straight dashed lines and shaded area) is described in detail in Ref. (28)

**Figure S4.** Frequency response of total elastic energy of a 2D glass with shear modulus distribution corresponding to **(a)** top left panel of Figure 4 and **(b)** top right panel of Figure 4. The marked frequency is the one chosen as excitation frequency for the strain energy maps. The VDoS Boson peak frequency $\omega_{\mathrm{BP}}$ is marked with the red line.
